# Effect of foliar sodium selenite fertilization on oxidative stress and productivity in *Olea europaea* L., biofortification and quality of extra virgin olive oil

**DOI:** 10.1002/jsfa.70164

**Published:** 2025-09-07

**Authors:** Kellyn Klein, Maria V. T. Mariano, Pietro P. M. Duran, Thais Posser, Jeferson L. Franco, Cimélio Bayer, Frederico C. B. Vieira

**Affiliations:** ^1^ Universidade Federal do Pampa, Campus São Gabriel—São Gabriel São Gabriel Brazil; ^2^ Universidade Federal do Rio Grande do Sul, Faculdade de Agronomia—Porto Alegre Porto Alegre Brazil

**Keywords:** selenium, olive tree, abiotic stress, antioxidant, hidden hunger, foliar fertilization

## Abstract

**Background:**

Fertilization of plants with selenium (Se) can enhance their resistance to abiotic stresses and improve human health and nutrition. However, Se fertilization in olive trees remains underexplored. This study evaluated the effect of foliar sodium selenite fertilization on leaf Se content, oxidative stress, olive tree productivity, biofortification of extra virgin olive oils (EVOO), and their physicochemical and antioxidant attributes in two mature ‘Arbequina’ olive orchards.

**Results:**

Although Se application did not affect fruit productivity, Se doses were related directly to total Se content in leaves. A slight increase in Se levels in EVOO was observed at the highest dose of sodium selenite applied. Lower levels of reactive oxygen species (ROS) were observed in leaves fertilized with 150 and 100 mg Se dm^−3^, after the first and second application, respectively. Application of Se had no clear effect on the physicochemical quality of olive oil or its antioxidant properties.

**Conclusion:**

Selenium application was effective in increasing Se concentration in leaves; however, it proved insufficient to enhance Se content in EVOO. Sodium selenite at the applied doses influenced oxidative stress parameters, exhibiting both antioxidant and pro‐oxidant effects over time. © 2025 The Author(s). *Journal of the Science of Food and Agriculture* published by John Wiley & Sons Ltd on behalf of Society of Chemical Industry.

## INTRODUCTION

The cultivation of the olive tree (*Olea europaea* L.) has been encouraged by the recognized health benefits of consuming olive oil.^1^ Regular consumption of extra virgin olive oil (EVOO) provides proven benefits for human health, associated with its anti‐inflammatory and antioxidant properties and its high polyphenol and monounsaturated fatty acid content.^2^, ^3^, ^4^ These benefits may be enhanced through targeted strategies.

The biofortification of olive products is a strategy to increase the intake of essential micronutrients, reducing the risk of nutritional deficiencies, or ‘hidden hunger’, which occur when diets fail to meet nutritional requirements for growth and development.^5^, ^6^ Iron, iodine, zinc, and selenium (Se) are essential for humans, and their deficiencies are major global nutritional challenges.^7^ Selenium stands out as particularly important for human health due to its ability to enhance antioxidant and anti‐inflammatory activity, and to help prevent cardiovascular diseases, certain types of cancer, and thyroid dysfunction.^5^, ^8^, ^9^, ^10^ These physiological functions are mediated by the action of at least 25 human selenoproteins, including glutathione peroxidases, selenoprotein P, thioredoxin reductases, and iodothyronine deiodinases.^10^, ^11^

Selenium deficiency and its adverse impact on human health have increased in recent decades.^10^ The World Health Organization (WHO) recommends a daily intake of 55 μg of Se per day for adults.^12^ However, it is estimated that a billion people worldwide have an inadequate intake of Se due to its low levels in the soil and consequently in food.^13^, ^14^ Socioeconomic factors also contribute, as significant Se sources are found in high‐value‐added foods such as meats, nuts, and seafood.^15^ Agronomic biofortification of Se is a key strategy to prevent and mitigate these deficiencies, offering an advantage over supplementation by converting inorganic Se into organic forms that are more bioavailable to humans.^16^

Besides its importance for human health, Se biofortification can support plant health and benefit olive growers by improving fruit yield in adverse environmental conditions and enhancing tree adaptation to extreme climate events. Although Se is not essential, it can benefit higher plants, particularly under adverse conditions.^17^, ^18^, ^19^ Although research on Se fertilization in olive trees is still incipient, some studies have demonstrated its potential to increase Se concentration and fruit yield, enhance the plant antioxidant defense system, reduce salt and drought stress, improve pollen viability under water deficit conditions, and alter the phenolic profile and pigment content of EVOO.^20^, ^21^, ^22^, ^23^, ^24^ These studies, conducted mainly in the Mediterranean Basin, typically applied Se as sodium selenate (Na₂SeO₄) using foliar spray, at doses ranging from 50 to 150 mg Se dm^−3^, depending on plant age and size.

Selenium enrichment of EVOO can provide nutritional benefits to consumers while enhancing the product's commercial value. Foliar fertilization with sodium selenate has been shown to enrich EVOO and table olives in terms of Se content, phenolic compounds, carotenoids, and chlorophylls, increasing their nutritional value and oxidative stability.^21^, ^24^, ^25^ This approach enhances qualitative and nutritional properties while potentially extending EVOO shelf life. However, the use of sodium selenite (Na₂SeO₃) in olive cultivation remains largely unexplored, despite its application in other crops.

This lack of research motivated the present study, which employed sodium selenite due to its greater accessibility for growers. Optimizing Se application to achieve an ideal dose–response based on the specific edaphoclimatic conditions of each region is essential to ensure beneficial outcomes for olive trees, especially considering that Se can become toxic within a narrow concentration range.^26^

This study aimed to evaluate the effects of selenium foliar fertilization using sodium selenite at different doses on total Se content, oxidative stress parameters, and productivity of mature olive trees, and to characterize the biofortification of extra virgin olive oils, their physicochemical qualities and antioxidant properties.

## MATERIAL AND METHODS

### Reagents and standards

Coomassie brillant blue G‐250, Triton X‐100, 2,7‐dichlorofluorescein diacetate (DCFH2‐DA), and 5,5′‐dithiobis‐(2‐nitrobenzoic acid) (DTNB) were purchased from Sigma‐Aldrich (St Louis, MO, USA). All other chemicals were of analytical grade.

### Experimental design

The field study was conducted on ‘Arbequina’ cultivar trees, from August 2022 to February 2023, in two adult olive groves, aged 7 and 6 years, located in São Gabriel (SG) (30° 20′S, 54° 21′ W) and Caçapava do Sul (CS) (30° 30′ S, 53° 29′ W), Rio Grande do Sul state, Brazil. Both areas have a humid subtropical climate (Cfa), according to the Köppen classification, with average annual rainfall of 1700 mm (SG) and 1745 mm (CS) and annual temperatures of 19.1 °C and 18.0 °C, respectively. The areas are classified as recommended for olive cultivation according to the agroclimatic zoning of Rio Grande do Sul.^1^ In June 2022, soil and leaf samples were taken from both sites for chemical analysis.

In the SG orchard, trees were planted 6 m apart between rows and 5 m apart within each row. The soil was Planosol as classified in the World Reference Base for Soil Resources/ Food and Agriculture Organization of the United Nations (WRB/FAO) system and Alfisol as classified in Soil Taxonomy. The soil had a silty loam texture in both the 0–20 cm and 20–40 cm layers. In the 0–20 cm layer, the soil contained 58.1% silt, 28.3% sand, and 13% clay, with moderate acidity (pH 6.0), 1.7% organic matter, 16 mg dm^−3^ exchangeable P, 93 mg dm^−3^ exchangeable K, 10.6 cmolc dm^−3^ exchangeable Ca, 5.1 cmolc dm^−3^ exchangeable Mg, 11 mg dm^−3^ available S, 2.6 mg dm^−3^ available Zn, 1.1 mg dm^−3^ available Cu, 4 mg dm^3^ available Mn, and 2.4 mg dm^−3^ available B. In the 20–40 cm layer, it contained 60.3% silt, 23.7% sand, and 16% clay, with pH 5.9, 1.5% organic matter, 8.9 mg dm^−3^ exchangeable P, 79 mg dm^−3^ exchangeable K, 10.9 cmolc dm^−3^ exchangeable Ca, 5.4 cmolc dm^−3^ exchangeable Mg, 11 mg dm^−3^ available S, 1.7 mg dm^−3^ available Zn, 0.9 mg dm^−3^ available Cu, 3 mg dm^−3^ available Mn, and 1.6 mg dm^−3^ available B. Due to poor drainage conditions, each row of olive trees was planted in raised beds of about 0.5 m height.

In the CS orchard, trees were planted 7 m apart between rows and 5 m apart within each row. The soil was a well‐drained soil classified as Regosol in the WRB/FAO system and Psamment in Soil Taxonomy. The soil had a sandy loam texture in both the 0–0.20 m and 0.20–0.40 m layers. In the 0–0.20 m layer, it contained 59.5% sand, 26.3% silt, and 14.2% clay, with moderate acidity (pH 5.5), 3.0% organic matter, 21 mg dm^−3^ exchangeable P, 159 mg dm^−3^ exchangeable K, 6.5 cmolc dm^−3^ exchangeable Ca, 3.4 cmolc dm^−3^ exchangeable Mg, 17 mg dm^−3^ available S, 2.5 mg dm^−3^ available Zn, 1.9 mg dm^−3^ available Cu, 21 mg dm^−3^ available Mn, and 4.4 mg dm^−3^ available B. In the 0.20–0.40 m layer, it contained 57.0% sand, 24.2% silt, and 18.8% clay, with pH 5.0, 2.1% organic matter, 7.0 mg dm^−3^ exchangeable P, 63 mg dm^−3^ exchangeable K, 3.8 cmolc dm^−3^ exchangeable Ca, 2.3 cmolc dm^−3^ exchangeable Mg, 25 mg dm^−3^ available S, 0.7 mg dm^−3^ available Zn, 1.2 mg dm^−3^ available Cu, 15 mg dm^−3^ available Mn, and 4.0 mg dm^−3^ available B.

Leaf nutrient concentrations differed between the two orchards. In the SG orchard, macronutrient levels were 1.5% N, 0.21% P, 1.0% K, 1.1% Ca, and 0.14% S, while micronutrients measured 118 mg kg^−1^ Cu, 25 mg kg^−1^ Zn, 393 mg kg^−1^ Fe, 13 mg kg^−1^ Mn, and 20 mg kg^−1^ B. In the CS orchard, leaves contained 1.4% N, 0.20% P, 1.2% K, 0.61% Ca, and 0.16% S, with micronutrients of 25 mg kg^−1^ Cu, 19 mg kg^−1^ Zn, 161 mg kg^−1^ Fe, 23 mg kg^−1^ Mn, and 19 mg kg^−1^ B. The concentration of Mn in the leaf tissue and the soil of the SG orchard was deficient. Both orchards had low Se content in the soil and leaf tissue, which was not detected by inductively coupled plasma optical emission spectrometry (ICP‐OES) (<0.849 mg Se kg^−1^).

The experimental design was in randomized blocks, with five treatments and five replications totaling 25 trees per site. Treatments applied Se doses of 0, 50, 100, 150 and 200 mg of Se dm^−3^, by foliar spray, using sodium selenite (Na_2_SeO_3_) diluted in water and 0.5% of Tec Oil wetting agent (surfactant). Each tree received 3 L of solution in each application. Se foliar fertilization was applied in August 2022 (6 months before commercial harvest) and in January 2023 (1 month before the commercial harvest).

### Selenium quantification

Selenium analysis in soil and leaf tissue was performed using ICP‐OES. Sample digestion and Se determination followed U.S. Environmental Protection Agency (EPA) methods. Methods 3050, 3051 A and 6010 D were used on the soil samples.^27^, ^28^, ^29^ Leaf tissue samples (0.2 g) were digested using microwave‐assisted wet digestion (MAWD) (UltraWave, Milestone, Italy) with nitric acid to ensure complete oxidation of organic matter. Selenium concentrations were measured following the PR‐Tb‐IN 014 method.^30^ Results were expressed in mg Se kg^−1^.

Olive oil samples (0.5 g) were digested using MAWD in 40 mL quartz vessels with 6 mL of sub‐boiled nitric acid. The digestion was carried out in a microwave system under a five‐step temperature program. After cooling, the digests were transferred to 50 mL polypropylene tubes and diluted to 20 mL with ultrapure water. Triplicates and one blank were prepared for each sample. Selenium was measured with inductively coupled plasma mass spectrometry (ICP‐MS) (Elan DRC II; PerkinElmer‐SCIEX, Thornhill, Canada). Results are expressed in μg Se g^−1^.

### Fruit yield

In February 2023, the commercial harvesting season fruit yield was evaluated by measuring the fresh fruit mass per tree. One kg of fruit per tree was kept at −4 °C for subsequent EVOO extraction.

### Sampling of plant material

Healthy, fully expanded leaves were collected from the middle third of the same‐year's branches at the four cardinal points of each tree. Sampling occurred 49 days after the first Se application and 36 and 39 days after the second Se application in CS and SG, respectively. The second leaf sampling coincided with the fruit harvest. In the laboratory, samples were stored at −80 °C until extraction and biochemical analyses.

### Biochemical analyses of plant tissue

#### Plant extract

The plant extract was prepared as described by Zhu *et al*.,^31^ with adaptations. Leaves were macerated in liquid nitrogen, homogenized in buffer solution (50 mM potassium phosphate pH 7.8, 1 mM ethylenediaminetetraacetic acid (EDTA) and 0.5% Triton X‐100) and filtered. The resulting solution was centrifuged at 13 000 × *g* for 20 min at 4 °C. The supernatant was stored at −80 °C until subsequent analyses.

#### Quantification of glutathione levels

Glutathione (GSH) total thiol levels were measured as described by Ellman^32^ with modifications. Supernatant (50 μL), 190 μL of 0.5 mol L^−^^1^ Tris/HCl pH 8.0 and 10 μL of 5 mM DTNB was added to a 96‐well plate and kept in darkness for 20 min. The reading was performed at 412 nm using a PerkinElmer EnSpire 2300 multimode microplate reader and the result was expressed in μmol GSH mg^−1^ of protein.

#### Lipid peroxidation estimation assay

Lipid peroxidation was measured using the method described by El‐Moshaty *et al*.^33^ with modifications. Vertical glass tubes were prepared with 150 μL of supernatant and 450 μL of buffer containing 20% trichloroacetic acid and 0.5% thiobarbituric acid. The tubes were heated in a water bath at 95 °C for 1 h 30 min and subsequently transferred to 96‐well plates for absorbance measurement at 532 nm using a PerkinElmer Enspire 2300 multilabel reader. Lipid peroxidation was expressed as nmol thiobarbituric acid reactive substances (TBARS) mg^−1^ protein.

#### Determination of reactive oxygen species

To determine reactive oxygen species (ROS) levels, oxidation of 2′‐7′‐dichlorofluorescein diacetate (DCFDA) was used as described by Lebel *et al*.,^34^ with modifications. Supernatant (20 μL) was incubated in 96‐well plates with 274 μL of 20 mM 4‐(2‐hydroxyethyl)‐1‐piperazineethanesulfonic acid (HEPES) buffer pH 7 and 6 μL of 10 mM 2′,7′‐dichlorofluorescein (DCF) reagent. Fluorescence emission from DCF was verified after 20 min at an excitation wavelength of 488 nm and emission wavelength of 530 nm. The values were normalized by the protein concentration in the samples and the result was expressed as total fluorescence.

### Fruit maturation index and oil yield

The fruit maturation index (MI) was evaluated per orchard, following an International Olive Council^35^ standard. Fruits from all replications were classified into categories 0–7 based on skin and pulp color, and the MI was then calculated using the following formula:

$$\mathrm{MI}=\frac{A^{*}0+B^{*}1+C^{*}2+D^{*}3+E^{*}4+F^{*}5+G^{*}6+H^{*}7}{100}$$

where the letters represent the numbers of fruits in each color category.

Due to laboratory extraction limits, only 0, 100 and 200 mg Se dm^−3^ doses were selected to obtain EVOOs for analyses. Fruits were crushed with a manual mill. Subsequently, malaxation was performed according to a method adapted from Miho *et al*.,^36^ with the fruits crushed for 30 min using a mixer‐type agitator, and kept in a water bath at 28 °C. The resulting paste was centrifuged at 1791 *g* for 10 min and the supernatant liquid fraction was separated and centrifuged two more times at 3168 *g* for 10 min, to separate the oil and aqueous phases. The EVOOs found were stored in amber glass bottles and kept in darkness at room temperature until required for analysis.

The oil yield was calculated using the volume of EVOO extracted per kg of olives sampled, using the calculation:

$$\text{oil yield}\;=\;\left(\left(\text{volume of oil extracted}/\mathrm{kg}\;\text{of fruit}\right)\;\times \;100\right)$$

expressed as a percentage.

### Physicochemical parameters of quality and antioxidant activity of EVOO

#### Quality index

The EVOO quality indices were evaluated by measuring peroxide value, free acidity, and specific ultraviolet extinction. Peroxide values were determined using an International Olive Council (IOC) method:^37^ samples were dissolved in a chloroform–acetic acid (3:2 v:v ratio), treated with potassium iodide, and titrated with sodium thiosulfate (0.01 mol L^−1^), using a starch solution as the indicator. The results were expressed in milliequivalents of oxygen per kg of olive oil (mEq O_2_ kg^−1^). Free acidity was evaluated using an IOC method,^38^ with diethyl ether–ethanol (1:1 v:v ratio) and titration with sodium hydroxide (0.1 mol L^−1^). The results were expressed as oleic acid percentages. Specific extinction in the ultraviolet at 232 and 270 nm was measured with a spectrophotometer (Agilent Technologies Cary 60 UV‐Vis Spectrophotometer, California, USA), by dissolving the samples in cyclohexane.^39^ All analyses were in triplicate.

#### Schaal oven test

Olive oil oxidative stability was evaluated using a modified Schaal oven test by measuring the peroxide index over time. Olive oil samples (100 g) were placed in 250 mL glass beakers and heated in an oven (70 °C ± 2 °C) without air circulation until the peroxide values reached 20 mEq O_2_ kg^−1^ or more, the legal limit for extra virgin olive oil.^40^, ^41^ Samples were analyzed at 12 h intervals during the first 48 h and subsequently at 24 h intervals until the last sample reached the established peroxide value, after a total of 96 h under accelerated oxidation conditions.

#### Chemical analyses of extra virgin olive oil

##### Obtaining the polar extract

Olive oil extracts were obtained by liquid–liquid extraction, following a method adapted from Nakbi *et al*.^42^ Olive oil (2.5 g) was mixed with 5 mL methanol/water (3:2) and 5 mL hexane, and homogenized for 10 min. The solution was centrifuged for 5 min at 5000 × *g*. The polar phase was removed with a syringe, filtered through a 0.45 μm nylon filter and used for analyses of phenolic compounds, flavonoids and *in vitro* antioxidant activity.

##### Total phenolic compounds and total flavonoids

Total phenolic compounds were quantified using a modified Folin–Ciocâlteu method.^43^ Polar olive oil extract (4 μL), distilled water (175 μL), Folin–Ciocalteu reagent (35 μL) and 15% sodium carbonate (70 μL) were added to a 96‐well plate. After 2 h, absorbance was measured at 760 nm using a PerkinElmer EnSpire 2300 multimode microplate reader. Results were calculated from a gallic acid standard curve (R^2^ = 0.9991) and expressed as mg gallic acid equivalents (GAE) per 100 g of polar extract.

Total flavonoid content was measured using a method described by Kumaran and Karunakaran,^44^ with modifications. Polar extract (150 μL) and 2% aluminum chloride (150 μL) were pipetted into the 96‐well plate. For the blank, 150 μL of distilled water and 150 μL of polar extract were pipetted. After 10 min, absorbance was read at 415 nm using a PerkinElmer EnSpire 2300 multimode microplate reader. The results were expressed as mg quercetin equivalents (QE) per 100 g of polar extract, based on a quercetin standard curve (R^2^ = 0.9984).

##### Total chlorophyll and total carotenoids

Pigments were determined following a method described by Minguez‐Mosquera *et al.*:^45^ olive oil (7.5 g) was dissolved in cyclohexane (25 mL), and absorption was measured at 670 nm for chlorophyll and 470 nm for carotenoids in a spectrophotometer (Agilent). The results are expressed as mg pheophytin kg^−1^ (total chlorophylls) and mg lutein kg^−1^ (carotenoids). Pigment concentration was expressed using the following equations:

chlorophyll=A670×106/613×100

carotenoids=A470×106/2000×100

where A670 and A470 represent absorbance at 670 and 470 nm, respectively.

##### *In vitro* antioxidant activity

Antioxidant activity was evaluated by the radical scavenging capacity of 2,2′‐azino‐bis(3‐ethylbenzothiazoline‐6‐sulfonic acid) (ABTS) in EVOO compounds, following a method adapted from Re *et al*.^46^ An ABTS stock solution (7 mM) was mixed with potassium persulfate (140 mM) and diluted 50‐fold for reactions. The polar extract was diluted in distilled water (900 mg mL⁻¹). In microplates, the following were pipetted: blank (250 μL distilled water), ABTS control (50 μL distilled water and 200 μL ABTS), and sample (2 μL extract, 48 μL distilled water, and 200 μL ABTS). Plates were incubated for 6 min in the dark, and absorbance was recorded at 734 nm using a PerkinElmer EnSpire 2300 multimode microplate reader. Blank absorbance was subtracted from all readings, and scavenging activity was calculated as:

$$\text{percentage scavenging}=\begin{array}{l} (\text{ABTS absorbance}-\;\text{sample absorbance})/\text{ABTS absorbance}\;\times \;100. \end{array}$$

The results are expressed as percentage scavenging at 900 mg mL^−1^ of extract.

Antioxidant activity was evaluated using the 1,1‐diphenyl‐2‐picrylhydrazyl (DPPH•) assay, which measures the ability of the sample to scavenge the stable DPPH radical, following a method described by Brand‐Williams^47^ with modifications. A DPPH stock solution (10 mM) was prepared in methanol and diluted to 900 μM for analysis. The polar extract was diluted to 900 mg mL⁻¹. The following were pipetted: in microplates: blank (300 μL methanol), DPPH control (270 μL methanol and 30 μL DPPH), and sample (10 μL extract, 260 μL methanol, and 30 μL DPPH). Plates were incubated in darkness for 45 min, and absorbance was measured at 517 nm using a PerkinElmer EnSpire 2300 multimode microplate reader. Blank absorbance was subtracted from all readings, applying the formula:

$$\text{percentage scavenging}=\begin{array}{l} \left(\text{DPPH absorbance}–\text{sample absorbance}\right)/\text{DPPH absorbance}\;\text{×}\;100 \end{array}$$

The result was expressed as percentage scavenging at 900 mg mL^−1^ of extract.

### Statistical analyses

Two‐way analysis of variance (ANOVA) (*P* < 0.05) was used to assess the effects of Se fertilization and block on fruit productivity and leaf biochemical parameters, with means compared using Tukey's test (*P* < 0.05). Regression analysis was applied to total Se content in leaves. All analyses and graphs, except principal component analysis (PCA), were performed in SigmaPlot v.11.0. The PCA was conducted in PAST v.4.17.

## RESULTS

### Selenium content in leaf tissue

**Figure 1:**
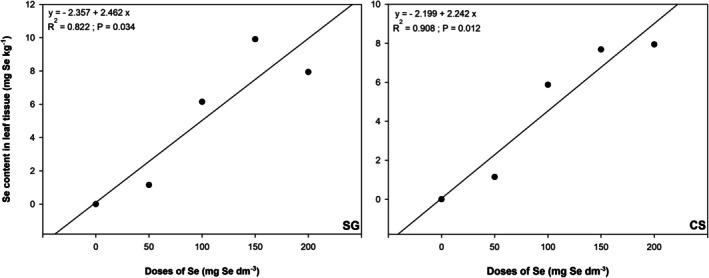
Relationship between the applied selenium dose (*x*) and leaf selenium content (*y*) in the SG (left) and CS (right) orchards, with linear regression fitted to the response. SG = São Gabriel. CS = Caçapava do Sul. Values expressed as averages of each treatment (*n* = 5).

In both orchards, an increase in the Se content of the leaves was observed with the applied Se doses, fitting a linear response function. The increases were relatively similar, as shown by the linear equations for each site. In the SG orchard (Fig. 1), the relationship between the applied Se dose (*x*) and the foliar Se content (*y*) is represented by the equation:

$$y=-2.357+2.462\;\times \;\left(R^{2}=0.822;\;P=0.034\right)$$

In the CS orchard (Fig. 1) the equation was:

$$y=-2.199+2.242\;\times \;\left(R^{2}=0.908;\;P<0.012\right)$$

Both the intercept and the angular coefficient were similar, indicating that distinct soil and climate conditions did not affect leaf Se content. The response was linear across the dose range used, with the highest Se dose resulting in the highest foliar Se content. Notably, the Se content in leaves from trees receiving the highest dose (200 mg per dm^−3^ of selenium) was similar to that in leaves from trees receiving 150 mg per dm^−3^ of selenium, suggesting stabilization at higher doses. The maximum Se content in the SG orchard was 9.9 mg Se kg^−1^ of dry mass (150 mg Se dm^−3^ treatment), whereas the highest in the CS orchard was 7.9 mg Se kg^−1^ of dry mass (200 mg Se dm^−3^ treatment). In trees without Se application, leaf Se content was below the ICP‐OES detection limit (0.849 mg Se kg^−1^) in all samples, reflecting the low Se content in the soils of the study sites.

### Fruit yield

**Figure 2:**
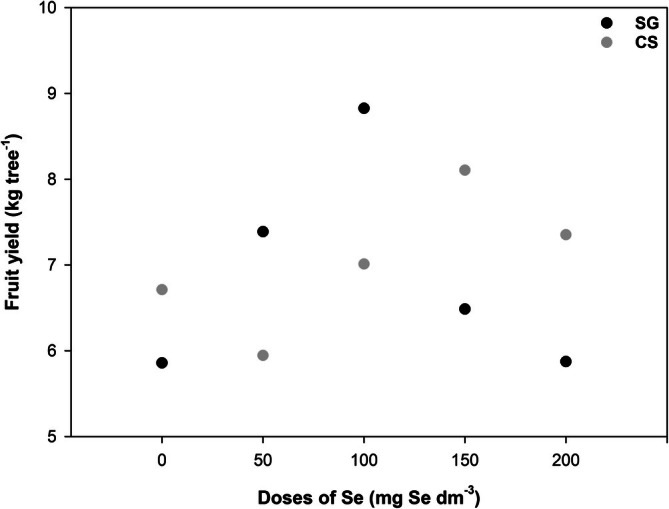
Fruit yield (kg per tree) of olive in SG (black dots) and CS (gray dots) orchards in relation to the foliar application of Se. SG = São Gabriel. CS = Caçapava do Sul. Values expressed as averages of each treatment in each site (*n* = 5).

In neither orchard was there a significant effect of Se doses on the fruit yield of olives (*P* > 0.05) (Fig. 2). Yields ranged from 5.8 to 8.8 kg per fruit tree, indicating a low yield typical of an ‘off’ year in the natural bearing of olive trees.

### Biochemical analyses of leaf tissue

In both leaf tissue analysis periods, a significant difference (*P* < 0.05) was observed only for ROS, in the CS orchard, indicated by 2′,7′‐dichlorodihydrofluorescein diacetate (DCF‐DA) fluorescence (Table 1). After the first application, samples with 150 mg Se dm^−3^ showed lower fluorescence (30.90) than the 0 mg Se dm^−3^ dose (49.61), indicating less ROS in Se‐fertilized plants.

**Table 1:** Analyses of glutathione (GSH) levels, lipid peroxidation, and reactive oxygen species in the leaf tissue of trees fertilized with different doses of selenium, after the first application of Se, in the SG and CS orchards

Dose of Se (mg dm^−3^)		0	50	100	150	200
Glutathione levels (μmol GSH mg protein^−1^)	SG	48.82 ns	51.21 ns	59.11 ns	63.23 ns	61.98 ns
	CS	48.67 ns	49.53 ns	47.48 ns	48.74 ns	49.19 ns
Lipid peroxidation (nmol TBARS mg protein^−1^)	SG	0.00185 ns	0.00173 ns	0.00183 ns	0.00193 ns	0.00181 ns
	CS	0.00257 ns	0.00214 ns	0.00206 ns	0.00215 ns	0.00240 ns
DCF‐DA fluorescence	SG	53.91 ns	55.85 ns	55.66 ns	57.82 ns	52.53 ns
	CS	49.61 b	47.35 ab	46.50 ab	30.90 a	38.71 ab

*Note*: Values expressed as means of repetitions (*n* = 5). Means followed by different letters in the rows represent significant differences between treatments (*P* < 0.05). Ns = no significant difference. 0 = 0 mg Se dm^−3^, 50 = 50 mg Se dm^−3^, 100 = 100 mg Se dm^−3^, 150 = 150 mg Se dm^−3^ and 200 = 200 mg Se dm^−3^. SG = São Gabriel. CS = Caçapava do Sul. DCF‐DA = 2′,7′‐dichlorodihydrofluorescein diacetate. GSH = glutathione.

Following the second Se application, samples from the 100 mg Se dm^−3^ treatment showed significantly lower mean fluorescence (10.02) than the 150 and 200 mg Se dm^−3^ doses (34.60 and 35.59, respectively), indicating an increase in ROS concentration in treatments with higher doses of Se (Table 2). However, a decrease in ROS was observed after the second application compared with the first, in both orchards (Tables 1 and 2). There was no significant difference in the levels of GSH and lipid peroxidation in leaf tissue between treatments, in both periods and olive groves (Tables 1 and 2).

**Table 2:** Analyses of glutathione (GSH) levels, lipid peroxidation, and reactive oxygen species in the leaf tissue of trees fertilized with different doses of selenium, after the second application of Se, in the SG and CS orchards

Doses of Se (mg dm^−3^)		0	50	100	150	200
Glutathione levels (μmol GSH mg protein^−1^)	SG	42.45 ns	43.16 ns	49.24 ns	46.26 ns	48.34 ns
	CS	49.04 ns	48.55 ns	49.67 ns	44.26 ns	53.14 ns
Lipid peroxidation (nmol TBARS mg protein^−1^)	SG	0.00165 ns	0.00160 ns	0.00167 ns	0.00172 ns	0.00176 ns
	CS	0.00138 ns	0.00133 ns	0.00131 ns	0.00142 ns	0.00146 ns
DCF‐DA fluorescence	SG	16.51 ns	25.92 ns	21.31 ns	22.57 ns	18.29 ns
	CS	24.54 ab	17.54 ab	10.02 a	34.60 b	35.59 b

*Note*: Values expressed as means of repetitions (n = 5). Means followed by different letters in the rows represent significant differences between treatments (*P* < 0.05). Ns = not significant differences. 0 = 0 mg Se dm^−3^, 50 = 50 mg Se dm^−3^, 100 = 100 mg Se dm^−3^, 150 = 150 mg Se dm^−3^ and 200 = 200 mg Se dm^−3^. SG = São Gabriel and CS = Caçapava do Sul. DCF‐DA = 2′,7′‐dichlorodihydrofluorescein diacetate. GSH = glutathione. TBARS = thiobarbituric acid reactive substances.

### Fruit maturation index and oil yield

The maturation index of the analyzed fruits was 2.14 in SG and 0.93 in CS, which was below the ideal harvesting range of 3 to 4, which ensures optimal balance between yield and sensory and quality characteristics of EVOO.^35^ The olive oil samples obtained from the SG orchard did not differ in terms of oil yield between the three treatments, resulting in 11% olive oil per sample. The olive oil yield of the CS samples was lower, reaching 8.33% for 0 mg Se dm^−3^ and 9.14% for 100 and 200 mg Se dm^−3^, due to the lower maturity of the fruits in this orchard (Table 3).

**Table 3:** Fruit maturation index and oil yield of the treatments selected for the extraction of olive oil samples from olive groves in SG and CS

	SG			CS		
Doses of Se (mg dm‐3)	0	100	200	0	100	200
Fruits maturity index	2.14			0.93		
Olive oil yield (%)	11.00	11.00	11.00	8.33	9.14	9.14

*Note*: 0 = 0 mg Se dm^−3^, 100 = 100 mg Se dm^−3^ and 200 = 200 mg Se dm^−3^.

Abbreviations: SG = São Gabriel and CS = Caçapava do Sul.

### Extra virgin olive oil

#### Selenium content in extra virgin olive oil

Only samples treated with 200 mg Se dm^−3^ yielded quantifiable values from ICP‐MS. The highest selenium content was detected in EVOO from SG (0.014 μg Se g⁻¹), whereas EVOO from CS contained 0.010 μg Se g⁻¹ (Table 4). Samples from the 0 and 100 mg Se dm^−3^ treatments were below the quantification limit (<0.008 μg Se g⁻¹).

#### Quality index of extra virgin olive oil

All EVOOs exhibited quality indices within the maximum limits established by Brazilian and international legislation,^40^, ^41^ indicating high quality (Table 4). Lower specific extinction at 232 nm was observed for oils from SG at 200 mg Se dm^−3^ and CS at 100 and 200 mg Se dm^−3^. At 270 nm, oils from SG 200 mg Se dm^−3^, CS 0 mg Se dm^−3^, and CS 100 mg Se dm^−3^ showed reduced absorption. No significant differences were found in free acidity and peroxide values among the treatments.

**Table 4:** Characterization of the physicochemical standards of olive oil quality and total selenium in extra virgin olive oil samples extracted from orchards in SG and CS

		SG			CS		
Doses of Se (mg dm^−3^)	0	100	200	0	100	200	Legislation^a^
Selenium content (μg Se g^−1^)	<0.008	<0.008	0.014 ± 0.002	<0.008	<0.008	0.010 ± 0.001	‐
Extinction UV (232 nm)	2.219 ± 0.00	2.045 ± 0.04	1.325 ± 0.02	2.043 ± 0.04	0.888 ± 0.09	0.861 ± 0.04	≤2.50
Extinction UV (270 nm)	0.136 ± 0.00	0.169 ± 0.02	0.053 ± 0.01	0.061 ± 0.00	0.082 ± 0.01	0.133 ± 0.05	≤0.22
Free acidity (% oleic acid)	0.46 ± 0.02	0.48 ± 0.02	0.44 ± 0.01	0.35 ± 0.01	0.46 ± 0.01	0.41 ± 0.02	<0.80
Peroxide index (mEq O_2_ kg^−1^)	3.90 ± 0.34	3.86 ± 0.09	3.63 ± 0.06	3.72 ± 0.14	2.99 ± 0.09	3.97 ± 0.03	≤20.00
Total phenol (mg GAE 100 g^−1^)	11.88 ± 0.01	11.88 ± 0.00	11.89 ± 0.01	11.88 ± 0.00	11.88 ± 0.00	11.88 ± 0.00	‐
Total flavonoids (mg QE. 100 g^−1^)	0.0504 ± 0.00	0.0560 ± 0.00	0.0546 ± 0.00	0.0462 ± 0.00	0.0560 ± 0.00	0.0574 ± 0.00	‐
Chlorophyll (mg pheophytin kg^−1^)	1.85 ± 0.00	1.85 ± 0.00	1.96 ± 0.02	3.49 ± 0.00	3.98 ± 0.02	3.60 ± 0.01	‐
Carotenoid (mg lutein kg^−1^)	1.97 ± 0.01	1.53 ± 0.00	1.50 ± 0.00	2.69 ± 0.00	3.01 ± 0.02	2.79 ± 0.02	‐
ABTS (% scavenging)	22.83 ± 0.00	18.37 ± 0.00	27.30 ± 0.00	14.17 ± 0.00	16.80 ± 0.00	12.60 ± 0.00	‐
DPPH (% scavenging)	4.146 ± 0.00	7.073 ± 0.00	10.244 ± 0.00	5.366 ± 0.00	1.951 ± 0.00	4.390 ± 0.00	‐

*Note*: Results expressed as means ± standard deviation of triplicates of each sample (n = 1). 0 = 0 mg Se dm^−3^, 100 = 100 mg Se dm^−3^ and 200 = 200 mg Se dm^−3^. SG = São Gabriel and CS = Caçapava do Sul. GAE = gallic acid equivalent. QE = quercetin equivalent. ABTS = ABTS radical scavenging activity. DPPH = DPPH radical scavenging activity.

^a^ BRASIL, CODEX = Brazilian legislation and Codex Alimentarius standarts for extra virgin olive oil.^40^, ^41^

**Table 5:** Principal component analysis (PCA) summary for the variables evaluated in olive oil

PC	Eigenvalue	% variance
1	4.44115	40.374
2	2.75318	25.029
3	2.03372	18.488

**Table 6:** Loadings of PCA showing relevance of variables on the axes

	PC 1	PC 2	PC 3
Selenium	0.22828	0.43509	−0.02538
UV (232 nm)	0.14819	−0.51679	−0.25863
UV (270 nm)	0.016731	−0.37008	0.45795
Free acidity	0.20452	−0.07693	0.54986
Peroxide index	0.16361	−0.29227	−0.08243
Total phenol	0.35702	0.36409	−0.1643
Total flavonoids	0.072829	0.28382	0.58889
Chlorophyll	−0.42572	0.25203	−0.05246
Carotenoid	−0.45281	0.1508	−0.04753
DPPH	0.41436	0.10583	−0.18304
ABTS	0.40781	0.068826	−0.03619

Abbreviations: DPPH = DPPH radical scavenging activity; ABTS = ABTS radical scavenging activity.

#### Total phenolic compounds and total flavonoids

Total phenolic compound levels were consistent across treatments, averaging 11.88 mg of GAE 100 g⁻¹ of polar extract in all EVOOs. Total flavonoid content did not differ between treatments, except for the CS 0 mg Se dm^−3^ sample, which was lower (0.0462 mg of QE. 100 g^−1^ of polar extract) than the other samples (Table 4).

#### Total chlorophyll and total carotenoids

Chlorophyll and carotenoid content was higher in EVOO from the CS orchard, reflecting lower fruit maturity. Within this orchard, the 100 mg Se dm^−3^ treatment exhibited the highest levels of both pigments. In contrast, SG samples had lower and less variable pigment levels, with highest carotenoid levels in the 0 mg Se dm^−3^ treatment and highest chlorophyll levels in the 200 mg Se dm^−3^ treatment (Table 4).

#### *In vitro* antioxidant activity

The antioxidant activity of EVOO extracts, as measured by ABTS radical scavenging, showed the highest inhibition in the order 200 > 0 > 100 mg Se dm^−3^ in the SG orchard, whereas in the CS orchard the order was 100 > 0 > 200 mg Se dm^−3^. Overall, SG oils exhibited higher inhibition than CS oils, indicating greater antioxidant capacity. For DPPH• radical scavenging, Se application enhanced antioxidant activity in SG EVOOs in a dose‐dependent manner, a trend not observed in CS. In both assays, the SG 200 mg Se dm^−3^ treatment showed the highest antioxidant values (Table 4).

#### Principal component analysis

**Figure 3:**
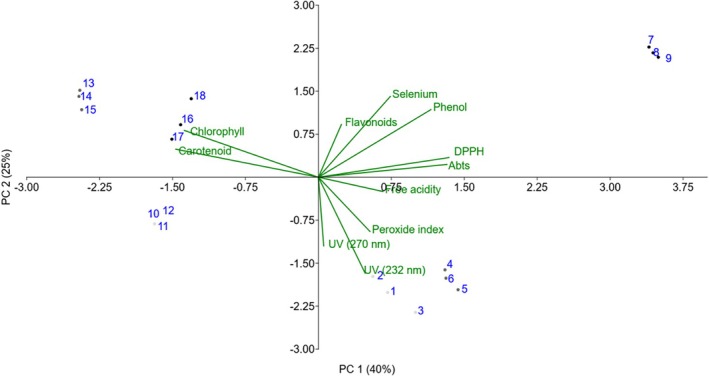
Principal component analysis of extra virgin olive oil samples.

Principal component analysis of the EVOO samples captured 83.89% of the total variance in the first three components (Table 5), with Table 6 showing the variables’ contributions to the axes. The first component explained 40% of the variance and was strongly associated with antioxidant activity, positively correlating with DPPH, ABTS, and phenolic compounds (Fig. 3), and negatively with carotenoids and chlorophyll. The second component explained 25% of the variance, showing positive correlations with Se, phenolic compounds, and flavonoids, and negative correlations with specific extinction at 232 and 270 nm.

#### Oxidative stability

**Figure 4:**
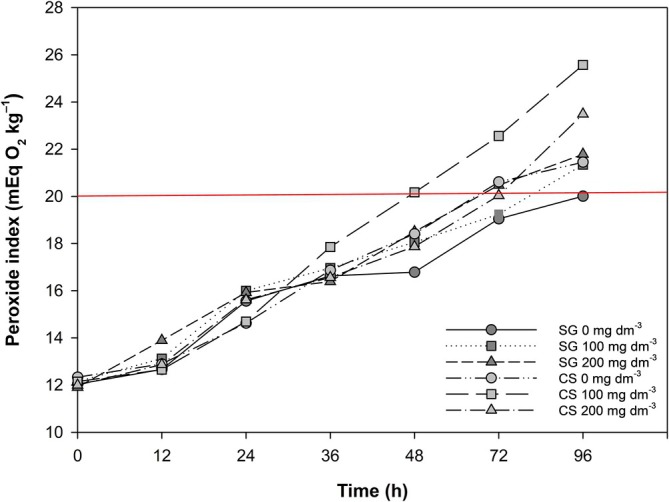
Peroxide index values of olive oil samples over 96 h, during the Schaal oven test. SG = São Gabriel. CS = Caçapava do Sul. Values are expressed as means of triplicates of each sample (*n* = 1).

Figure 4 shows the peroxide index of EVOOs subjected to different Se treatments in SG and CS. The CS 100 mg Se dm^−3^ sample exhibited the lowest oxidative stability, reaching 20 mEq O₂ kg^−1^ within 48 h. Oils from CS at 0 and 200 mg Se dm^−3^ and SG at 200 mg Se dm^−3^ reached this value within 72 h. The highest oxidative stability was observed in SG oils at 0 and 100 mg Se dm^−3^, which maintained lower peroxide levels after 96 h of accelerated oxidation.

## DISCUSSION

Selenium content in leaf dry mass increased linearly with Se dose, reaching 9.9 mg Se kg^−1^ in SG (150 mg Se dm^−3^) and 7.9 mg Se kg^−1^ in CS (200 mg Se dm^−3^), confirming efficient absorption of foliar‐applied sodium selenite. Other studies reported increases in Se content in olive leaves with 50 and 150 mg Se dm^−3^ of sodium selenate, averaging 0.026 and 0.036 mg Se kg^−1^ of fresh mass.^20^ No visual symptoms of Se toxicity, such as leaf chlorosis or necrosis, were observed at any dose.

Regarding oxidative stress in leaves, Se fertilization reduced ROS levels significantly in CS after the first Se application, with the 150 mg Se dm^−3^ treatment showing lower fluorescence than the control without Se. Proietti *et al*.^20^ also noted decreased MDA levels following foliar Se application. After the second application, ROS levels declined in both orchards, although the reduction was not strictly dose‐dependent. In CS, 100 mg Se dm^−3^ resulted in lower fluorescence than the higher doses (150 and 200 mg Se dm^−3^), suggesting a pro‐oxidant effect of sodium selenite. Yildiz *et al*.^48^ observed increased oxidative stress, through the accumulation of ROS and lipid peroxidation in maize seedlings with excess Se.

No significant differences in fruit productivity were observed across Se doses and Se application did not reduce the trees’ productive potential. The apparently higher fruit yield at intermediate Se doses requires confirmation in high fruit‐yield years because this study coincided with a low‐yield (‘off’) year. Olive trees naturally exhibit alternate bearing, which is influenced by multiple biotic and abiotic factors. The low yield may have masked the effect of Se, as the yields in the orchards that were studied can surpass 40 kg of fruit per tree in ‘on’ years (personal communication). Some studies have reported increased fruit yield following Se application. Olive trees fertilized with 50 and 150 mg Se dm^−3^ by soil^49^ and 100 mg Se dm^−3^ by foliar spray^24^ showed higher olive productivity under conditions of water stress, in comparison with unfertilized trees. In both cases, sodium selenate was used as the chemical source of Se.

Although Se application increased Se content in leaves proportionally to the doses, this pattern was not observed in the EVOO. Selenium was only detected at the highest dose, which increased the Se content slightly in EVOO. To reach the recommended daily intake of 55 μg Se day^−1^ for adults, approximately 3928 g of biofortified olive oil with 0.014 μg Se g^−1^ would be necessary. This makes the strategy entirely impractical, suggesting that olive oil biofortification may not be a suitable approach to effectively increase selenium intake in the human population. Evaluating the efficiency of foliar selenium application using sodium selenate for biofortification, D'Amato *et al*.^24^, ^29^also reported low Se concentration in EVOO. However, the levels they observed were higher than those found in the current study, with values of 0.956 and 0.529 μg Se g^−1^ in EVOO at application rates of 150 and 100 mg Se dm^−3^, respectively. This can be attributed to the Se source, solution volume applied, application timing, plant age, and soil conditions. Selenium biofortification of food crops is effective in other species, including rice,^17^ wheat,^14^ carrots,^50^ and coffee.^51^ However, research specifically on olive oil is still lacking.

The evaluated EVOOs demonstrated good quality, with peroxide, free acidity, and specific UV extinction values below the limits established for EVOO. Lower UV absorption was observed with 100 and 200 mg Se dm^−3^, indicating reduced secondary oxidative compounds. Extra virgin olive oils from CS had lower absorption values at both wavelengths, and higher chlorophylls and carotenoids content, possibly due to lower fruit maturity. Free acidity and peroxide values did not vary between treatments. D'Amato *et al*.^49^ also reported no influence of Se on these indices.

The PCA of the EVOO samples highlighted key variations in physicochemical characteristics. The first component was strongly associated with antioxidant activity, showing positive correlations with DPPH, ABTS, and phenolic compounds. These antioxidant compounds contribute to the olive oil's capacity to neutralize free radicals, directly influencing stability and health benefits.^52^, ^53^ Conversely, the negative association with carotenoids and chlorophyll suggests that samples with higher antioxidant activity tend to have lower concentrations of pigments. In the second principal component, higher Se content was linked to increased phenolic compounds and flavonoids, together with lower specific extinction at 232 and at 270 nm. This pattern suggests that samples with higher levels of selenium and antioxidant compounds exhibit lower UV absorption, indicating a lower degree of oxidation.

The Schaal oven test results did not reveal a clear effect of Se on oxidative stability. The CS treatment with 100 mg Se dm^−3^ reached the threshold of 20 mEq O₂ kg^−1^ – the legal limit for EVOO^40^, ^41^ – faster than the other treatments. Oils from the 200 mg Se dm^−3^ treatment reached this limit after 72 h, whereas those from SG 0 and 100 mg Se dm^−3^ showed greater stability, requiring up to 96 h to reach the peroxide value. Bonissoni *et al*.^54^ reported lower oxidative stability in Arbequina EVOO from Santa Catarina, also in southern Brazil, with the threshold reached after only 24 h, suggesting that the EVOO in the present study had higher quality and resistance to oxidation.

Oxidative stability is a key measure of oil quality as it reflects susceptibility to oxidative degradation, the primary cause of deterioration.^55^ The peroxide value, the most representative parameter for measuring oxidation in virgin olive oils, indicates the level of primary oxidation products.^56^ Selenium may exert a pro‐oxidant effect, accelerating oxidation and thereby reducing oxidative stability in oils from Se‐treated plants. However, this effect was inconsistent and cannot be confirmed, as the SG treatment with 100 mg Se dm^−3^ showed higher oxidative stability, comparable with the control.

Selenium foliar fertilization effectively increased total Se content in leaves in proportion to the doses tested. However, only a slight increase in Se levels was detected in olive oil at the highest dose, 200 mg Se dm^−3^. These findings indicate that biofortification of EVOO through this method is not an efficient strategy for enhancing human selenium intake. Application of sodium selenite influenced oxidative stress markers in the plants over time, confirming its dose‐dependent role in modulating both antioxidant and pro‐oxidant metabolic pathways. Fruit productivity was unaffected by the applied doses, and no visual symptoms of Se toxicity were observed in any plant. Selenium application also showed no clear effect on the physicochemical quality or antioxidant properties of the resulting olive oil.

Future research should test different doses and application frequencies to better assess the potential for increasing Se content in olive oil, as well as possible improvements in its physicochemical quality and oxidative stability.

## FUNDING INFORMATION

This work was supported by the Universidade Federal do Pampa and the Coordenação de Aperfeiçoamento de Pessoal de Nível Superior (CAPES).

## Data Availability

The data that support the findings of this study are available from the corresponding author upon reasonable request.
